# Isowighteone attenuates vascular calcification by targeting HSP90AA1-mediated PI3K-Akt pathway and suppressing osteogenic gene expression

**DOI:** 10.3389/fbioe.2025.1636883

**Published:** 2025-08-20

**Authors:** Yuanxi Mo, An Jin, Wanzi Hong, Jiahui Peng, Rui Yang, Qiqi Song, Yaoxin Liu, Yuqi Cheng, Wing-Tak Wong, Qian Huang, Lei Jiang, Zhaoyan Xu, Ning Tan

**Affiliations:** ^1^ Department of Cardiology, Guangdong Cardiovascular Institute, Guangdong Provincial People’s Hospital, Guangdong Academy of Medical Sciences, Guangzhou, China; ^2^ School of Pharmaceutical Sciences, Hunan University of Medicine, Huaihua, China; ^3^ Department of Cardiology, The First People Hospital of Foshan, Foshan, China; ^4^ School of Medicine South China University of Technology, Guangzhou, China; ^5^ Department of Applied Biology and Chemical Technology, The Hong Kong Polytechnic University, Hong Kong, Hong Kong SAR, China; ^6^ Hubei Key Laboratory of Biomass Fibers and Eco-Dyeing & Finishing, Department of Chemistry and Chemical Engineering, Wuhan Textile University, Wuhan, China

**Keywords:** vascular calcification, network pharmacology, isowighteone, *Ficus hispida* L.f, PI3K-AKT

## Abstract

**Background:**

Isowighteone, an isoflavonoid compound derived from *Ficus hispida* L.f. (*F. hispida*, Moraceae), has demonstrated significant anti-inflammatory properties in prior studies. However, its anti-inflammatory role in vascular calcification is unclear.

**Object:**

We investigated the efficacy of isowighteone in the treatment of vascular calcification, explored its potential mechanism, and determined whether isowighteone is a safe and effective treatment.

**Methods:**

In this study, we isolated three natural compounds and evaluated their efficacy using *in vitro* calcification models through CCK-8 assays, Alizarin Red staining, and calcium quantification. The key targets of Isowighteone were identified via network pharmacology and molecular docking analyses. The anti-calcification effect of Isowighteone was further assessed in a mouse model of vascular calcification. Alizarin Red staining, calcium quantification, and immunofluorescence were employed to evaluate its therapeutic potential. Additionally, quantitative real-time PCR (qRT-PCR) and Western blot were used to examine the mRNA and protein expression levels of osteogenic markers. The impact of Isowighteone on the HSP90AA1/PI3K/Akt signaling pathway in vascular calcification was also investigated using Western blot analysis.

**Results:**

Alizarin red staining and Calcium quantification experiments demonstrated that Isowighteone reduces aortic vascular calcification in mice and decreases calcification levels in Human aortic smooth muscle cells (HASMCs). Network pharmacology and molecular docking analysis reveals the HSP90AA1 protein as the specific target of isowighteone in HASMCs which PI3K-Akt is pivotal regulatory signaling pathway in this mechanism. Additionally, this study proved Isowighteone downregulated osteogenic gene expression in HASMCs, thereby inhibiting cellular calcification and preventing the process of VC by *in vivo* study, as evidenced by qRT-PCR and Western blot.

**Conclusion:**

Isowighteone demonstrates significant therapeutic potential by effectively downregulating the expression of osteogenic genes, alleviating vascular calcification, and suppressing the HSP90AA1/PI3K/Akt signaling pathway, thereby improving pathological conditions associated with vascular calcification. These above results not only elucidate isowighteone as a novel therapeutic agent against VC through selective suppression of osteogenic differentiation but also position this phytochemical as a clinically candidate for VC management.

## 1 Introduction

Vascular calcification (VC) is a common pathological condition linked to multiple pathologies, including chronic kidney disease (CKD), vascular injury, atherosclerosis, and aging (Zhang et al., 2021; Turner et al., 2024). Characterized by osteogenic transdifferentiation of vascular smooth muscle cells, VC drives calcium-phosphate deposition, impairing vascular contractility and accelerating atherosclerotic plaque progression. Currently, no clinically approved therapies can reverse vascular calcification (Durham et al., 2018), highlighting the urgent need for targeted therapies to address this pathology.

VC is a chronic, progressive pathological process characterized by vascular wall thickening, stiffening, and ectopic mineralization, ultimately leading to reduced arterial compliance and increased cardiovascular risk. This pathological cascade initiates with endothelial injury, which activates inflammatory responses and promotes inflammatory cell infiltration (Cui et al., 2024; Soehnlein et al., 2025; Yao et al., 2025). This establishes a pro-inflammatory and osteogenic microenvironment that drives pathological calcification progression. Within this microenvironment, Vascular smooth muscle cells (VSMCs) undergo osteogenic phenotypic transition through exposure to inflammatory mediators, culminating in pathological vascular remodeling and ectopic calcification of vascular walls. Calcifying VSMCs demonstrate marked upregulation of key osteogenic markers, including bone morphogenetic protein 2(BMP2), alkaline phosphatase (ALP), and runt-related transcription factor 2 (RUNX2). This pathological transition is orchestrated by activated signaling pathways such as Notch, Wnt/β-catenin, and NF-κB (Li et al., 2024; Wang et al., 2024; Xu et al., 2024). Given the intricate pathogenic mechanisms driving VC, the identification of effective therapeutic targets to attenuate its pathological progression is critical for managing associated cardiovascular pathologies.

Natural medicinal plants have emerged as valuable sources for treating cardiovascular diseases, offering advantages such as improved efficacy and reduced toxicity (Xu et al., 2020; Zhang et al., 2023; Guo B. et al., 2024). Among these, the genus Ficus is a well-regarded medicinal plant widely reported for its therapeutic effects on cardiovascular conditions (Al-Shabibi et al., 2022; Sahu et al., 2024). The flavonoid components of Ficus exhibit excellent anti-inflammatory activity, which are key contributors to its medicinal properties. Specifically, *F. hispida* has been clinically employed in traditional medicine for ulcer management; inflammation modulation; and fever reduction, with emerging evidence supporting its pharmacological basis (Zhang et al., 2018; Cheng et al., 2020). From *F. hispida*, three isoflavonoids with significant anti-inflammatory effects have been isolated in our laboratory. We test them for validate its anti-inflammatory function by *in vitro* experiment. Moreover, one of the isoflavonoids, as called isowighteone may hold potential for the treatment of VC, which is closely related to inflammation processes.

Network pharmacology acting as an interdisciplinary approach integrating bioinformatics and systems biology which provides a robust analytical framework for elucidating drug-disease interactions through multi-target and multi-pathway mechanisms (Nogales et al., 2022; Zhang et al., 2023). As natural products represent over 50% of modern therapeutics with proven clinical efficacy (Pal and Shukla, 2003), network pharmacology serves as a critical tool for systematically mapping phytochemical constituents and their polypharmacological effects in medicinal plants. This study employs network pharmacology to identify and evaluate isowighteone’s potential mechanisms in treating VC. Through KEGG pathway enrichment analysis, we identified calcification-related signaling hubs, followed by molecular docking simulations to validate isowighteone’s binding affinity with core targets. Experimental validation using HASMCs models under calcifying conditions revealed its inhibition of calcium deposition and downregulation of osteogenic markers. These findings provide a mechanistic framework for developing therapeutics targeting VSMC phenotypic switching and ectopic mineralization in VC.

This study revealed that osteoblastic differentiation of cells plays a pivotal role in the progression of valve calcification. Isowighteone was found to inhibit osteogenic differentiation of HASMCs through the HSP90AA1/PI3K/Akt signaling pathway. These findings underscore the potential therapeutic value of Isowighteone in treating VC.

## 2 Materials and methods

### 2.1 Plant material

The fruits of *F. hispida* used in this study were collected in Wenchang city of Hainan Province, People’s Republic of China, in September 2022 (latitude 19.5614° N, longitude 110.8023° E). A voucher specimen (20220901) was deposited in the Herbarium of Materia Medica, School of Pharmaceutical Sciences, Hunan University of Medicine. Isowighteone, 3′-(3-methylbut 2-enyl)biochanin A, and myrsininone A was isolated from the collected fruits. The purity of these isolated compounds was assessed using HPLC and was found to be greater than 98%.

### 2.2 Isolation of human aortic smooth muscle cells

This study was approved by the Research Ethics Committee of Guangdong Provincial People’s Hospital and was performed in accordance with the Declaration of Helsinki. The enrolled patients included 8 males and 3 females, with a mean age of 62.5 years (range: 53–72 years). The etiology of aortic dissection among participants primarily included hypertension-induced degeneration, and atherosclerosis. HASMCs were isolated from the aorta of a patient with aortic dissection. Briefly, the median membrane of the aorta was removed and the cells were incubated with 1 mg/mL trypsin (Gibco, 12605-010) for 10 min. The cells were washed with HBSS buffer (Hyclone, SC30588.01). Aortic tissue was digested in 250 U/mL collagenase type II solution (Worthington, 47D17411A) at 37 ° C for 7 h. The resulting cells were resuspended in α-MEM containing 10% fetal bovine serum (Gibco, 16000-044), 100 U/mL penicillin, and 100 mg/mL streptomycin (HyClone, SH40003.01.) SV30010) in growth medium and plated on a 25 cm^2^ flask coated with 0.25 μg/cm^2^ type I collagen (Gibco, A1048301). Two to four generations of cells were used in this study.

### 2.3 Induction of HASMCs *in vitro* calcification

HASMCs were inoculated into 6-well plates at a density of 1.0 × 105 cells/well and cultured with growth medium. As mentioned above, calcification was simply induced by culturing HASMCs to fusion and treating with a control (1.0 mM Pi/1.8 mM Ca) or a calcifying medium (50 μg/mL ascorbate/2.5 mM Pi/2.7 mM Ca) for 7 days. Pi was prepared using NaH2PO4/Na2HPO4 as solvent and pH = 7.4. To evaluate the effect of isowighteone on calcifying HASMCs *in vitro*, a certain concentration (50 μM) of isowighteone was added to the medium. The medium was changed every 2/3 days.

### 2.4 Animal study

Male C57BL/6 mice, 6–8 weeks old, purchased from Rueger Biology Co., LTD. The 45 mice were randomly divided into three groups with 15 mice in each group. To establish a mouse calcification model, high doses of vitamin D (5*105 IU/kg/day) were injected subcutaneously into the abdominal cavity of each mouse for 3 consecutive days and continued to be fed for 4 days. After the vitamin Dinjection, mice in the model group were given normal saline by gavage, and mice in the treatment group were given 50 mg/kg isowighteone by gavage daily for 4 consecutive days. On day 7, at the end of the study, the mice were euthanized under 2% pentobarbital anesthesia and the mouse aorta was excised. The control group received no treatment, and the aorta was collected on day 7. The research plan of this animal experiment was approved by the Animal Ethics Committee of Guangdong Provincial People’s Hospital, Guangzhou, China. All animal procedures were performed in accordance with the recommendations of the National Science and Technology Commission of the People’s Republic of China in the Guide to the Care and Use of Laboratory Animals.

### 2.5 Cell viability analysis

Cell viability was assessed by CCK-8 assay (Biosharp, BS350A). Specifically, HASMCs was plated in 96-well plates at a density of 2 × 10^4 cells per well and then incubated overnight in a 5% CO^2^ atmosphere at 37 °C to allow cell attachment and stabilization. After incubation, the effects of the compound on cell viability at different doses (50 μM, 100 μM and 150 μM) were measured at 24 and 48 h time points. Before detection, 10 μL CCK-8 solution was added to each well and incubated for 2 h. The optical density of each well was then quantitatively measured at a wavelength of 450 nm, providing a direct reading of cell viability for the treatment condition.

### 2.6 Immunofluorescence staining

To assess RUNX2 expression, HASMCs were cultured with control or calcified media in the presence or absence of 50 μM isowightone for up to 7 days. Cells were fixed, infiltrated with 0.5% Triton X-100(Beyotime Biotechnology, P0013B) and treated with anti-RUNX2 (1:500, proteintech, 20700-1-AP) at 4 °C overnight. After frozen sectioning of isolated mouse aorta, antigen repair was performed and antiRUNX2 (1:500, Proteintech, 20700-1-AP) was treated overnight at 4 °C. After washing with PBS, Alexa Fluor^®^488 anti-rabbit antibody (1:1,000, Invitrogen, A11008)were incubated in blocking buffer at 37 °C in the dark for 2 h. Fluorescence signals were detected under a Leica DMRB fluorescence microscope (Leica SP8) with a DAPI-stained glass mask.

### 2.7 Quantitative real-time polymerase chain Reaction (qRT-PCR)

Total RNA was extracted from HASMCs using Trizol (Invitrogen, 1596026) according to the manufacturer’s instructions. Quantification and reverse transcription of RNA was performed using HiScript III RT SuperMix for qPCR(+gDNA wiper) (Vazyme, R323-01). qRT-PCR was performed in a QuantStudio 5 real-time system (Life technologies) using ChamQ Universal SYBR qPCR Master Mix (Vazyme, Q711-01). Each PCR was repeated in triplicate. All gene expression data were calculated using 2^−ΔΔCT^ and normalized to β-actin. β-actin was used as the housekeeping gene after confirming its stable expression under both control and calcifying conditions. The control value is expressed as 1 to represent the exact fold change value for each gene of interest. Primer sequences of the target genes are summarized in Supplementary Table S1.

### 2.8 Western blot

HASMCs was harvested using a radioimmunoprecipitation (RIPA) lysis buffer (Beyotime Biotechnology, P0013B) containing protease and phosphatase inhibitor (Thermo, A32961). Western blot was performed. An equal amount of protein lysate was separated on an SDS-polyacrylamide gel and transferred to a polyvinylidene fluoride (PVDF) membrane. The membrane was incubated with primary antibodies overnight at 4 °C: anti-HSP90AA1 (1:2,000, Proteintech, 13171-1-AP), anti-RUNX 2 (1:2,000, Proteintech, 82636-2-RR), anti-BMP2 2 (1:1,000, Proteintech, 66383-1-Ig), anti-p-Akt (1:2,000, Proteintech, 66444-1-Ag), anti-Akt (1:2,000, Proteintech, 10176-2-AP), anti-MSX2 (1:2,000, Proteintech, 68550-1-Ig), anti-PI3K(1:1,000, Cell Signaling Technology, 4292), anti-p-PI3K(1: 1,000, Cell Signaling Technology, 3011S), anti-beta-actin (1:2,000, Proteintech, 66009-1-Ig), followed by anti-mouse (1:400, Cell Signaling Technology, 7076S) or anti-rabbit (1:400, Cell Signaling Technology, 7074S) secondary antibodies coupled to the membrane with horseradish peroxidase (HRP) were incubated at room temperature for 1 h. The immune complex was visualized using a chemiluminescent Western blot Substrate (Millipore, WBKLS0500). Semi-quantitative assessment of band intensity was performed using ImageJ software (National Institutes of Health).

### 2.9 Collection of targets associated with isowightone and vascular calcification

The Search Tool for Interacting Chemicals (STITCH) and the SwissTargetPrediction database were used to predict interacting molecular targets associated with isowightone. The collection of targets associated with VC was obtained from Genebank.

### 2.10 Protein-protein interaction (PPI) network

PPI data of SO-related molecular targets were collected using STRING online database, Cytoscape 3.2.1 software was run to visualize the PPI relationship network.

### 2.11 GO and KEGG pathway enrichment analysis

Gene Ontology (GO) functional annotation and Kyoto Encyclopedia of Genes and Genomes (KEGG) pathway enrichment analysis were performed using the DAVID database (https://david.ncifcrf.gov/). This comprehensive analysis included the three major categories of GO: biological process (BP), cellular component (CC), and molecular function (MF). Pathways and GO terms with p < 0.05 were considered significantly enriched, and false discovery rate (FDR) correction was applied to adjust for multiple comparisons. The genes most significantly associated with enriched GO terms and KEGG pathways were prioritized based on their logP values.

### 2.12 Molecular docking

Structural data for the two primary target proteins were obtained from the Universal Protein Resource (UniProt: https://www.uniprot.org/) and the Research Collaboratory for Structural Bioinformatics Protein Data Bank (RCSB PDB: https://www.rcsb.org/).Simultaneously, the three-dimensional structures of two key small-molecule ligands were retrieved from PubChem (https://pubchem.ncbi.nlm.nih.gov/). All receptor proteins were processed and visualized using PyMOL version 2.5.2. Prior to molecular docking with AutoDock Vina version 1.1.2, a grid box was defined around the active sites of the receptors to ensure accurate interaction analysis. The docking results were evaluated based on binding affinity, and the conformation with the lowest binding energy was selected as the most favorable ligand–receptor binding mode.

### 2.13 Synthesis of isowighteone–Cy5 conjugate

Isowighteone (10 mg) was dissolved in anhydrous dimethyl sulfoxide (DMSO, 1 mL).Separately, Cy5-NHS ester (Lumiprobe, United States) was dissolved in DMSO (10 mM stock). The two solutions were mixed at a 1:1.5 M ratio (isowighteone:Cy5-NHS), and 3 equivalents of DIPEA (N,N-diisopropylethylamine) were added to catalyze the reaction. The mixture was stirred under nitrogen at room temperature, protected from light, for 12 h. After the reaction, the product was purified using C18 reverse-phase HPLC, and the conjugate was identified by UV–Vis spectroscopy (showing peaks at ∼290 nm and ∼650 nm) and LC-MS to confirm the expected molecular weight. The final compound was lyophilized and stored at −20 °C in the dark.

### 2.14 Cellular uptake

HASMCs were seeded in 24-well plates containing coverslips and cultured to approximately 70% confluence. Cells were incubated with either free Cy5(500 nM) or an equimolar concentration of Isowighteone-Cy5 conjugate in serum-free medium for 12 h at 37 °C. After incubation, cells were washed with PBS, fixed with 4% paraformaldehyde for 15 min, and stained with DAPI. Coverslips were mounted on slides, and fluorescence images were acquired using a confocal laser scanning microscope (excitation/emission for Cy5: 650/670 nm).

### 2.15 *In vivo* imaging

Male C57BL/6J mice (8–10 weeks old) were randomly divided into two groups and injected via tail vein with either free Cy5 or Isowighteone-Cy5(1 nmol in 100 µL PBS). Whole-body fluorescence imaging was performed using an *in vivo* imaging system (IVIS Spectrum, PerkinElmer) at 0, 1, 2, and 4 h post-injection.

### 2.16 Statistical analysis

Data are presented as mean ± SD. Statistical significance was determined by one-way ANOVA followed by Dunnett’s multiple comparisons using GraphPad Prism (GraphPad software version 7.0, San Diego, CA, United States). P < 0.05 was considered statistically significant.

## 3 Results

### 3.1 Biochemical analysis of isowighteone and related compounds

**FIGURE 1:**
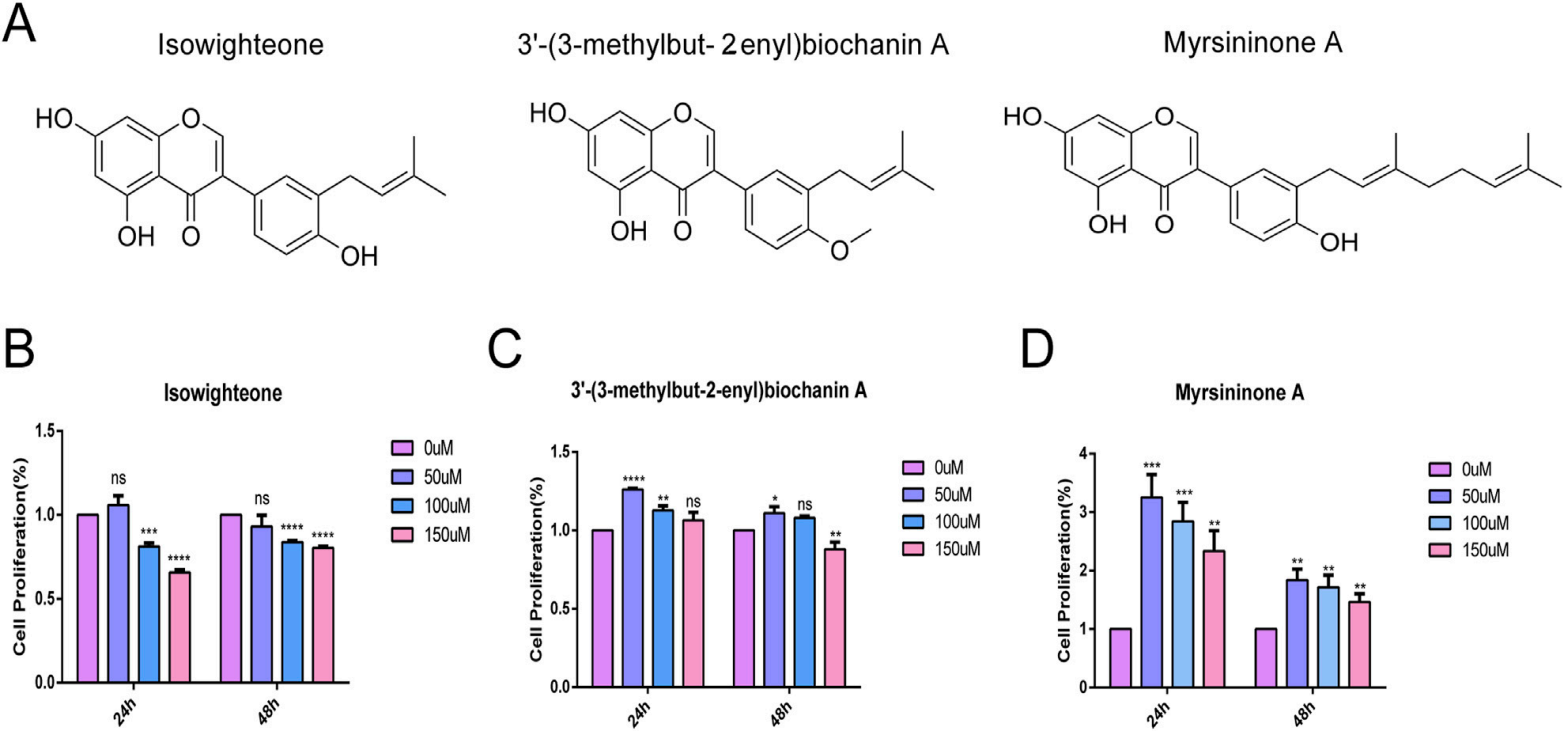
Biochemical analysis of three compounds. **(A)** Structure of three natural compounds. **(B–D)** Effects of three compounds on HASMCs at concentrations of 0 μmol/L, 50 μmol/L, 100 μmol/L and 150 μmol/L for 24 h and 48 h, respectively. All experiments were performed in triplicate (n = 3). p < 0.05 (*), p < 0.01 (**), p < 0.001 (***), and p < 0.0001 (****).

Three isoflavonoids, isowighteone, 3′-(3-methylbut 2-enyl)biochanin A (Biochanin A) (hereafter referred to as Biochanin A), and myrsininone A (Figure 1A), were isolated from the fruit of *F. hispida*. CCK-8 assays demonstrated that all three compounds exerted minimal cytotoxicity on HASMCs at a concentration of 50 μM over 24 and 48 h (Figures 1B–D), establishing 50 μM as the optimal concentration for subsequent experiment.

### 3.2 Isowightone inhibits calcification in HASMCs

**FIGURE 2:**
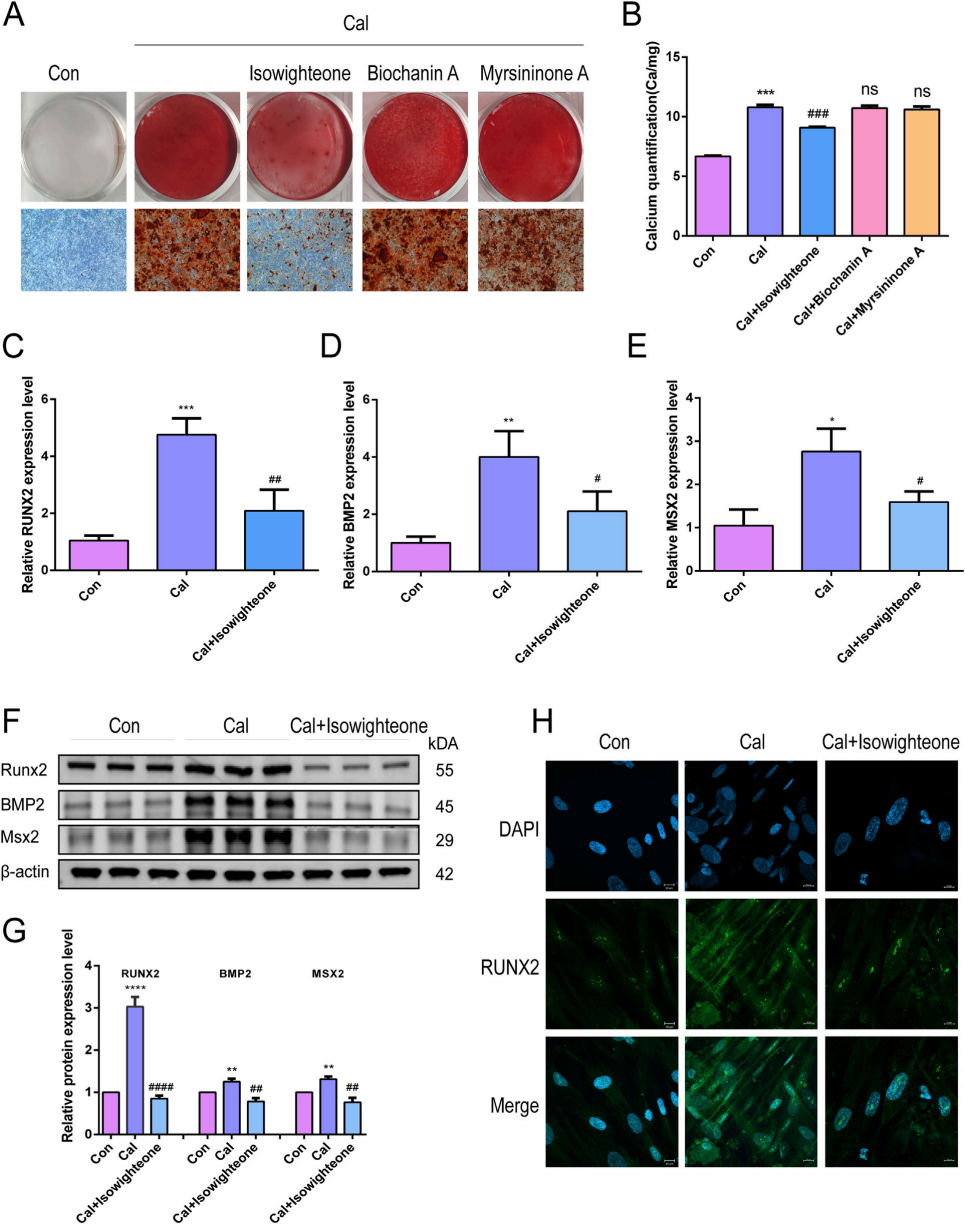
Isowightone inhibits calcification in HASMCs. **(A)** Alizarin red staining was used to evaluate the effects of three compounds on calcified HASMCsin vitro. **(B)** The effects of three compounds on calcification of HASMC *in vitro* were quantitatively evaluated by Calcium quantification. **(C)** The effect of isowighteone on RUNX2 expression was evaluated by qRT-PCR. **(D)** The effect of isowighteone on BMP2 expression was evaluated by qRT-PCR. **(E)** The effect of isowighteone on MSX2 expression was evaluated by qRT-PCR. **(F,G)** The effect of isowighteone on osteogenic differentiation was detected by Western blot. **(H)** The expression of RUNX2 was evaluated by immunofluorescence, Scale bar = 10 μm* is compared with control group, # is compared with calcification group. The therapeutic concentration of all three compounds was 50 μM.All experiments were performed in triplicate (n = 3). p < 0.05 (*), p < 0.01 (**), p < 0.001 (***), and p < 0.0001 (****).

Exposure of HASMCs to 50 μM isowighteone significantly reduced calcium deposition after 7 days, as evidenced by alizarin red staining and calcium quantification (Figures 2A,B). In contrast, 3′-(3-methylbut-2-enyl)biochanin A and myrsininone A did not notably affect calcification levels. Furthermore, isowighteone attenuated the upregulation of osteogenic markers RUNX2, BMP2, and MSX2 at both mRNA and protein levels (Figures 2C–G). Immunofluorescence imaging corroborated the reduction in nuclear RUNX2 expression upon isowighteone treatment (Figure 2H). Collectively, isowighteone exhibits superior efficacy in inhibiting calcification and osteogenic gene expression compared to the other tested compounds.

### 3.3 Collection of VC-related therapeutic targets

**FIGURE 3:**
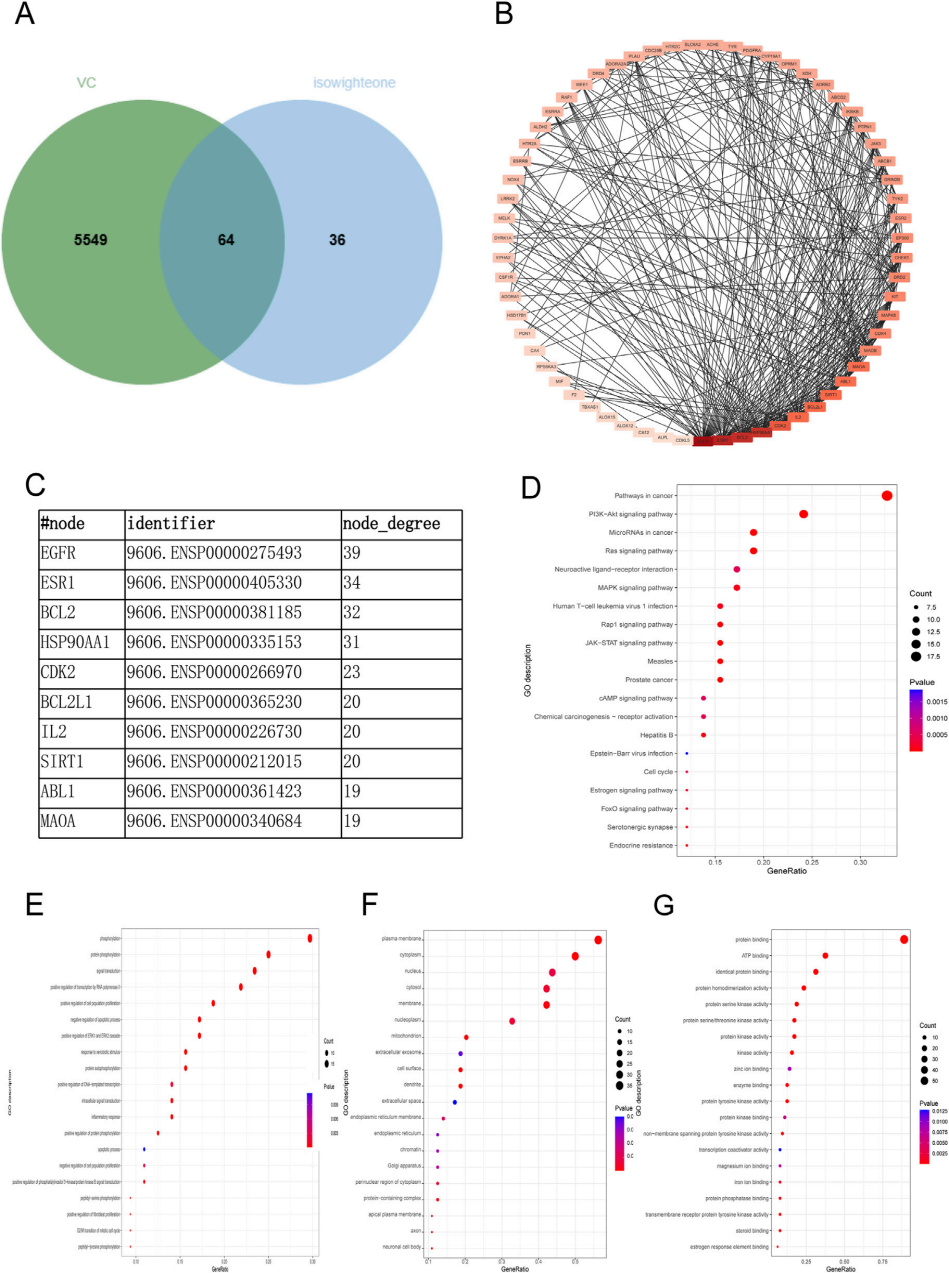
Network pharmacological analysis of VC-related therapeutic targets. **(A)** Isowighteone and VC are common targets. **(B)** Common target to obtain PPI networks. **(C)** The top 10 nodes of PPI networks. **(D)** KEGG scatter plot was used to analyze the pathway of isowighteone in the treatment of VC. **(E–G)** Bubble diagram of BP, CC, MF. The Y and x-axes represent the full names of process and gene ratios, respectively. The color and size of each bubble is based on a P-value and a gene count, respectively.

A comprehensive target analysis identified 5,604 VC-related targets from the GeneCards database and 17 from the OMIM database, totaling 5,613 unique targets after deduplication. Intersection with 100 isowighteone-related targets revealed 64 overlapping targets (Figure 3A; Supplementary Table S2). Based on the candidate protein targets of isowighteone,we constructed the PPI network, which contained 64 nodes and 337 edges (Figure 3B; Supplementary Table S3). The top 4 proteins were considered as the critical molecular targets mediating the anti-VC effects of isowighteone. It was found that EGFR, ESR1, BCL2 and HSP90AA1 were separately linked to other 39,34,32 and 31 targets, respectively (Figure 3C).

To further verify the biological characteristics of 64 isowighteone targets on VC, we performed enrichment analysis of KEGG pathways to explore potential pathways. The 20 most enriched pathways are shown in Figure 3D. The KEGG pathways are detailed in Supplementary Table S4. These enriched targets are associated with a variety of pathways, including primarily those associated with inflammation and angiogenesis. Among the 64 overlapping targets, 26 closely interacted with pathways associated with the cancer pathway, the PI3K−Akt signaling pathway, MicroRNAs in cancer pathway and the RAS pathway (Figure 3D). Details of KEGG analysis are provided in Supplementary Table S4. Next, we performed GO enrichment analysis to clarify their biological processes. The top 20 entries based on the value of p. The GO enrichment analysis provided insights into the roles of intersecting targets across the domains of BP, CC, and MF. In the BP domain, the genes were primarily involved phosphorylation, protein phosphorylation, signal transduction, and positive regulation of transcription by RNA polymerase II (Figure 3E). Within the CC domain, the predominant genes were associated with plasma membrane, cytoplasm, and nucleus (Figure 3F). For the MF domain, the key genes were involved in protein binding, ATP binding and identical protein binding, among other molecular functions (Figure 3G). Details of GO analysis are provided in Supplementary Tables S5–S7. These findings suggest that isowighteone may exert its anti-VC effects through modulation of these key signaling pathways.

### 3.4 Effects of isowighteone on the HSP90AA1-PI3K/Akt pathway *in vitro*

**FIGURE 4:**
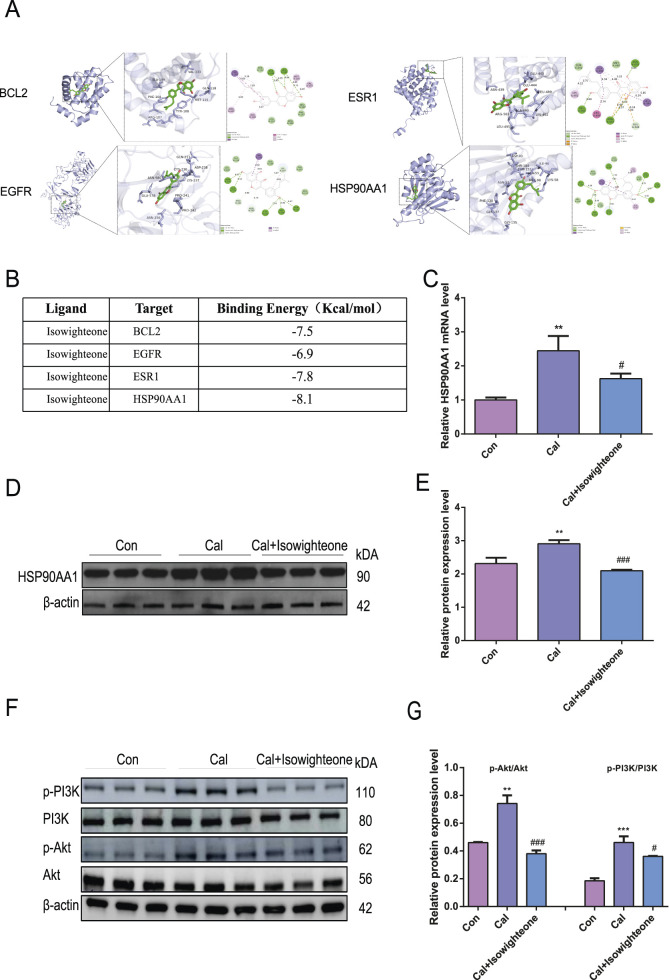
Effects of isowighteone on the HSP90AA1-PI3K/Akt pathway *in vitro*. **(A)** Molecular docking results of BCL2(PBD ID: 2XA0), EGFR (PBD ID: 5u3), ESR1(PBD ID: 1A52) and HSP90AA1(PBD ID: 1BYQ) with isowighteone. The ligand has a green rod-like structure, the receptor protein is purple, and the van der Waals force is generated by green, non-linking amino acids around the compound. Hydrogen bonds are green and occur linking amino acids; Hydrophobic interactions (including π-* interactions) are purple, resulting in linked amino acids; The electrostatic force is orange, and the linkage of amino acids occurs. **(B)** The bingding energy of isowighteone binding to key target molecules. **(C)** The effect of isowighteone on HSP90AA1 expression was evaluated by qRT-PCR. **(D,E)** Western blot analysis was performed to assess the effect of isowighteone on HSP90AA1 protein expression. **(F,G)** The figure shows that isowighteone processes the phosphorylation levels of PI3K and Akt in descendants’ vascular smooth muscle cells. The therapeutic concentration of isowighteone was 50 μM. * is compared with control group, # is compared with calcification group. All experiments were performed in triplicate (n = 3). p < 0.05 (*), p < 0.01 (**), p < 0.001 (***), and p < 0.0001 (****).

Molecular docking studies were performed to evaluate the binding affinity of isowighteone to its core target genes. The main targets identified due to their centrality in the network include BCL2 (PDB ID: 2XA0), EGFR (PDB ID: 1YY9), ESR1 (PDB ID: 1A52), and HSP90AA1, (PDB ID: 1BYQ) as protein receptors. Isowighteone, as a natural compound, acts as a ligand in the validation of molecular docking procedures (Figure 4A). The docking results showed that the binding energies of these compounds and the target were all below −6.90 kcal/mol, indicating that there was a strong binding effect between them (Figure 4B). It is noteworthy that isowighteone interacts particularly strongly with HSP90AA1, showing the most favorable binding energy. The binding pattern between isowighteone and the central gene is shown in the figure, which shows multiple interaction types at the active site, including van der Waals forces, hydrogen bonds, hydrophobic interactions, and electrostatic forces.Subsequently, qRT-PCR and Western blot analyses confirmed that isowighteone significantly downregulated HSP90AA1 expression in calcifying cells (Figures 4C–E). These findings suggest that isowighteone may exert its anti-VC effects through modulation of HSP90AA1. Given the pivotal role of the PI3K/Akt pathway in VC, Western blot analyses revealed that isowighteone reduced the phosphorylation levels of PI3K and Akt without altering their total protein expression (Figures 4F,G). These results implicate isowighteone as an inhibitor of the PI3K/Akt pathway, thereby attenuating VC.

### 3.5 Isowighteone inhibits vascular calcification *in vivo*

**FIGURE 5:**
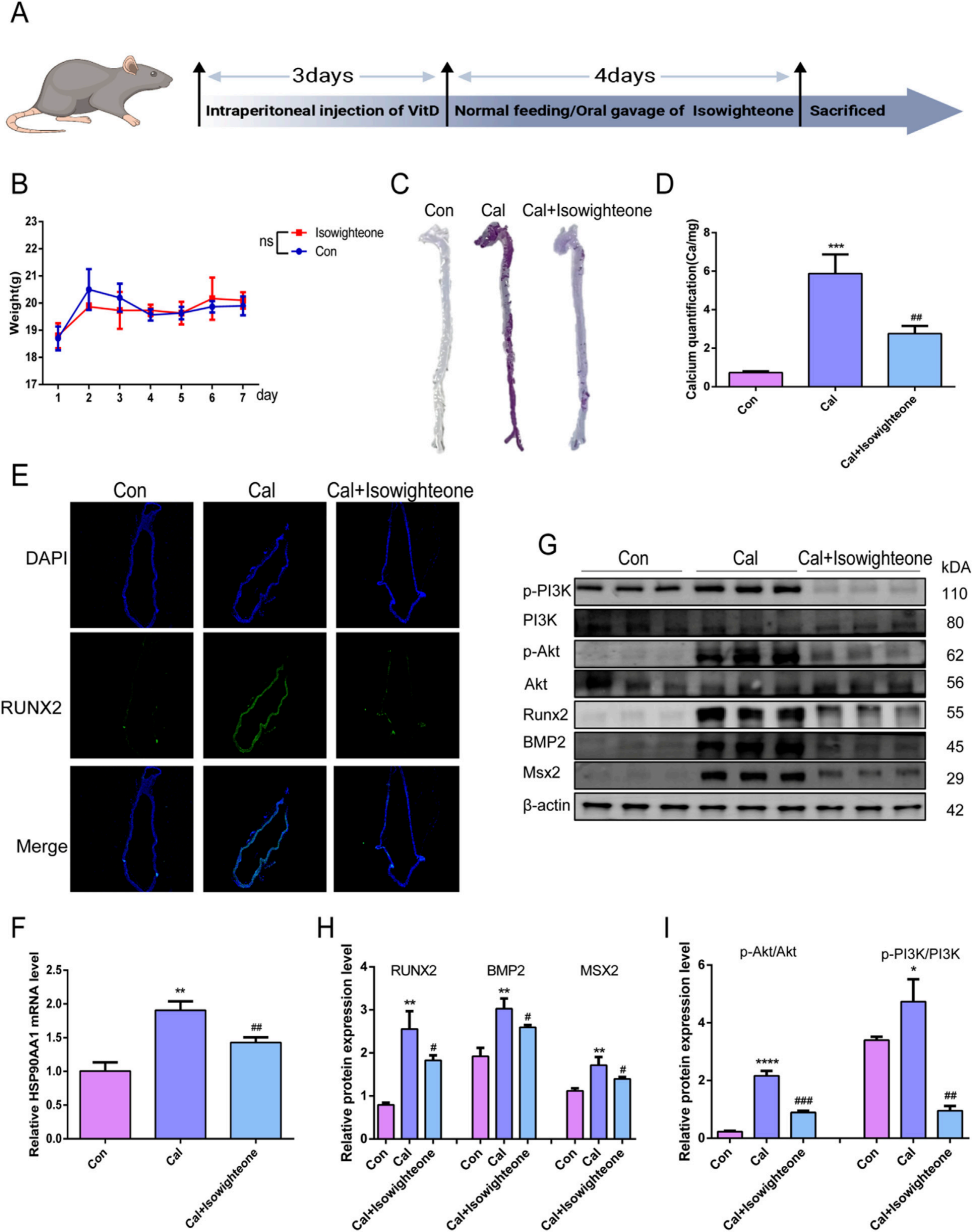
Isowighteone inhibits VC *in vivo*. **(A)** Temporal Framework for Calcification Modeling and Therapeutic Intervention in Mice. **(B)** Effect of isowighteone on body weight in mice. **(C)** The effect of isowighteone on aortic calcification in mice was evaluated by alizarin red staining. **(D)** The effect of isowighteone on aortic calcification in mice was evaluated by Calcium quantification. **(E)** The expression of isowighteone on osteogenic differentiation gene was detected by immunofluorescence assay in mouse aorta, Scale bar = 100 μm. **(F)** The effect of isowighteone on HSP90AA1 expression was evaluated by qRT-PCR. **(G–I)** Western blot analysis was performed to assess the effect of isowighteone on RUNX2、BMP2、MSX2、p-PI3K、PI3K、p-Akt、Akt protein expression *in vivo*. In each independent experiment, three mice were included in each group. All experiments were performed in triplicate (n = 3). p < 0.05 (*), p < 0.01 (**), p < 0.001 (***), and p < 0.0001 (****).

To investigate the anti-calcification effects of isowighteone *in vivo*, we established a mouse model of VC through high-dose vitamin D administration (Figure 5A). Oral administration of isowighteone did not significantly affect body weight, suggesting a favorable safety profile (Figure 5B). Alizarin red staining and quantitative calcium analysis revealed that isowighteone markedly attenuated aortic calcification in the VC model (Figures 5C,D). Furthermore, fluorescence staining demonstrated a significant reduction in RUNX2 expression in the aortic tissues of treated mice (Figure 5E). Given the critical role of osteogenic genes in calcification, we evaluated the expression of RUNX2, BMP2, and MSX2, and found that isowighteone effectively suppressed their levels *in vivo* (Figure 5G). These findings collectively indicate that isowighteone significantly reduces calcification in the VC model.

To elucidate the underlying mechanisms, we examined the expression of HSP90AA1 in mouse aortic smooth muscle cells. qRT-PCR confirmed that isowighteone downregulated HSP90AA1 expression (Figure 5F). Additionally, considering the pivotal role of the PI3K/Akt signaling pathway as a downstream effector, we assessed its activity via Western blot (Figures 5G–I). The results demonstrated that isowighteone suppresses PI3K/Akt signaling transduction. Together, these *in vivo* results underscore the therapeutic potential of isowighteone in mitigating VC.

### 3.6 The biodistribution of isowighteone *in vivo*

**FIGURE 6:**
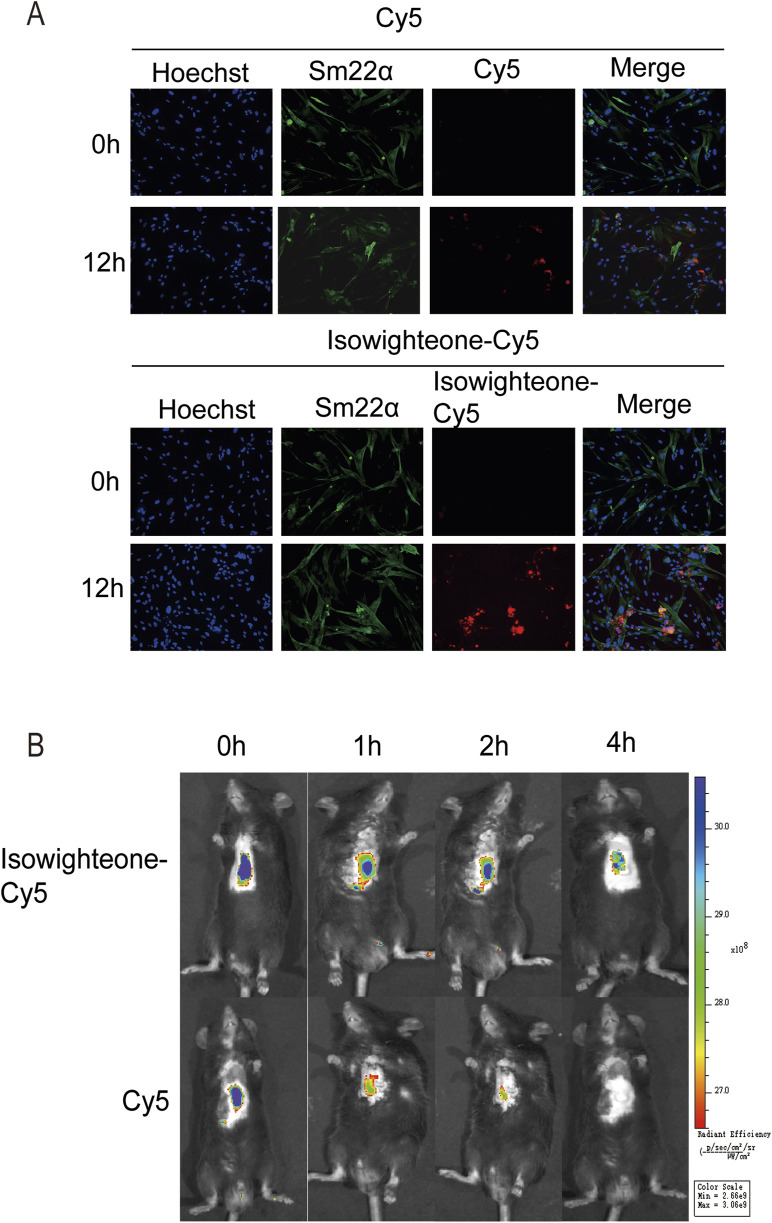
Cellular uptake and *in vivo* biodistribution of Isowighteone-Cy5. **(A)** Representative fluorescence images of HASMCs after incubation with free Cy5 or Isowighteone-Cy5 for 12 h, showing enhanced intracellular uptake of the isowighteone conjugate. **(B)** *In vivo* fluorescence imaging of mice following tail vein injection of free Cy5 or Isowighteone-Cy5. Fluorescence signals were recorded at 0, 1, 2, and 4 h post-injection, demonstrating prolonged circulation time of Isowighteone-Cy5 in cardiac and perivascular regions. All experiments were performed in triplicate (n = 3).

To investigate the biodistribution of isowighteone *in vivo*, we synthesized a Cy5-labeled isowighteone (Isowighteone-Cy5) conjugate. Following incubation with HASMCs for 12 h, immunofluorescence analysis revealed a markedly higher intracellular fluorescence signal in the Isowighteone-Cy5 group compared to cells treated with free Cy5, indicating enhanced cellular uptake of isowighteone (Figure 6A).

For *in vivo* evaluation, Isowighteone-Cy5 or free Cy5 was intravenously administered via tail vein injection in mice. Fluorescence imaging at 0, 1, 2, and 4 h post-injection showed that Isowighteone-Cy5 exhibited slower clearance compared to free Cy5. Notably, the conjugate exhibited evident accumulation around vascular structures, demonstrating that isowighteone is capable of accumulating in perivascular tissues *in vivo* (Figure 6B).

## 4 Discussion

VC is an active, bone-like pathological process that primarily occurs in the arterial vessel wall, particularly in the medial layer and VSMCs regions. It is characterized by calcium salt deposition and vascular stiffening (Leopold, 2015). VC is a major risk factor for various cardiovascular diseases, such as atherosclerosis, chronic kidney disease, and diabetes, and is closely associated with cardiovascular events and increased mortality (Ghosh et al., 2020; Kaur and Singh, 2022; Li et al., 2022). The pathogenesis of VC is highly complex, involving multiple processes including cellular phenotypic transition, inflammatory responses, oxidative stress, apoptosis, and mineral metabolism disorders (Lee et al., 2020). Currently, there is no specific targeted therapy for VC; clinical management mainly relies on the control of underlying conditions, which has limited efficacy in halting VC progression. Therefore, there is an urgent need to develop novel therapeutic strategies. In recent years, natural products have shown significant potential in modulating VC-related signaling pathways, particularly by regulating calcium and phosphate metabolism, inhibiting osteogenic gene expression, and alleviating inflammation and oxidative stress (Lim et al., 2021; Liu et al., 2021; Yang et al., 2023). Mouse models of VC induced by high-phosphate diets or excessive vitamin D3 are considered classical experimental models, as they closely mimic the mechanisms of VC in humans. In this study, we investigated the effects of Isowighteone on VC. The results demonstrated that Isowighteone effectively inhibited the formation of calcified plaques, reduced vascular calcium content, suppressed osteogenic phenotypic transformation of VSMCs, and thereby intervened in the progression of VC.

*Ficus hispida* L.f. has garnered significant attention due to its rich medicinal value and a wide range of pharmacological activities, including anti-inflammatory, antioxidant, antimicrobial, and analgesic effects (Cheng et al., 2020). It has been extensively used in traditional medicine to treat various ailments such as gastrointestinal disorders, skin diseases, and respiratory infections. The roots, leaves, and fruits of *F. hispida* contain numerous bioactive compounds, including flavonoids, terpenoids, and alkaloids, which have shown remarkable effects in alleviating inflammatory responses and modulating immune functions (Ali and Chaudhary, 2011). For instance, *F. hispida* extracts have been demonstrated to effectively reduce mucosal damage in experimental rat models of gastric ulcer by inhibiting gastric acid secretion, enhancing antioxidant defenses, and promoting mucosal healing (Rao et al., 2008). An increasing number of studies also suggest that *F. hispida* may exert its immunomodulatory and anti-inflammatory effects by regulating key signaling pathways such as NF-κB and MAPK, highlighting its potential as a natural therapeutic source for chronic inflammatory (Jia et al., 2020).

Among the natural products derived from *F. hispida* L.f., flavonoids have attracted considerable attention due to their diverse structures and broad-spectrum biological activities. For example, flavonoids such as quercetin and kaempferol are abundant in the leaves and fruits of *F. hispida* and exhibit remarkable anti-inflammatory, antioxidant, antimicrobial, and cytoprotective properties (Carullo et al., 2017; Sun et al., 2019; Alizadeh and Ebrahimzadeh, 2022). These flavonoid compounds are increasingly recognized for their potential roles in chronic inflammatory diseases such as ulcers, and metabolic conditions (Figueiredo et al., 2023; Miao et al., 2024). The synergistic actions of flavonoids in immunomodulation, free radical scavenging, and suppression of inflammatory pathways offer new perspectives for their application in the treatment of VC (Vera Saltos et al., 2015; Chen et al., 2016; Zeinali et al., 2017). Given their favorable biosafety profile and multi-target regulatory properties, flavonoid derivatives derived from *F. hispida* may provide valuable pharmacological insights and candidate compounds for therapeutic research on VC and other immune-inflammatory diseases.

We isolated three flavonoid compounds from *F. hispida* L.f., namely, Isowighteone, 3'-(3-methylbut-2-enyl)biochanin A, and Myrsininone. In previous studies, all three compounds demonstrated notable anti-inflammatory activity, indicating their pharmacological potential. In the present study, we systematically evaluated the effects of these natural products on VC and found that Isowighteone exhibited a significant inhibitory effect on vascular calcium deposition, suggesting its promising therapeutic potential in the treatment of VC.

In this study, network pharmacology was employed to further explore the potential mechanisms by which the natural compound Isowighteone exerts its therapeutic effects against VC. Through network pharmacology analysis, the protein–protein interaction (PPI) network identified four key targets—BCL2, EGFR, ESR1, and HSP90AA1—that may play important roles in the development and progression of VC. Molecular docking and dynamics simulations further confirmed the strong binding affinity of Isowighteone to these proteins, with HSP90AA1 showing particularly notable binding capability. Moreover, by investigating the interactions among compounds, targets, and signaling pathways, molecular biology approaches further validated that Isowighteone effectively inhibits the PI3K/Akt signaling pathway.

HSP90AA1 is a molecular chaperone that plays a critical role in regulating various signaling pathways and is essential for maintaining cellular homeostasis, stress responses, and protein stability (Zuehlke et al., 2015). Studies have shown that HSP90AA1 can activate the PI3K/Akt signaling pathway by stabilizing its downstream effectors, including phosphoinositide 3-kinase (PI3K) and protein kinase B (Akt), thereby participating in key biological processes such as cell proliferation, apoptosis, and inflammation (Guo Y. et al., 2024). In the context of VC, abnormal activation of the PI3K/Akt pathway is recognized as a critical mechanism promoting the phenotypic transition of vascular smooth muscle cells into osteoblast-like cells. Specifically, Akt activation upregulates osteogenic genes such as RUNX2 and BMP2, thereby enhancing calcium deposition and accelerating vascular wall calcification (Chen et al., 2019; Gao et al., 2023). Importantly, a previous study on warfarin-induced VC demonstrated that HSP90AA1 contributes to vascular calcification by modulating the PI3K/Akt signaling pathway, further supporting its role in VC pathogenesis and its identification as a core target in our network pharmacology analysis (Zhang et al., 2025).

In summary, our study systematically elucidates the potential mechanism by which Isowighteone alleviates VC. Isowighteone inhibits the expression of HSP90AA1, thereby blocking the activation of the PI3K/Akt signaling pathway mediated by this chaperone protein. As a result, it suppresses the osteogenic transdifferentiation of VSMCs and significantly reduces calcium deposition. Concurrently, we also observed a downregulation in the expression of VC-related osteogenic markers such as RUNX2 and BMP2, further confirming its anti-calcification effect.

## 5 Conclusion

This study demonstrates that isowighteone, a natural compound isolated from *F. hispida*, exerts anti-VC effects by inhibiting the HSP90AA1-PI3K/Akt signaling pathway. Both *in vitro* and *in vivo* experiments corroborate the network pharmacology predictions, highlighting isowighteone’s potential as a therapeutic agent for individuals diagnosed with VC. Further investigations are warranted to explore additional pathways and validate the clinical applicability of isowighteone in treating VC.

## Data Availability

The original contributions presented in the study are included in the article/Supplementary Material, further inquiries can be directed to the corresponding authors.

## Abbreviations

VC, Vascular calcification; PPI, Protein-protein interaction; KEGG, Kyoto Encyclopedia of Genes and Genomes; GO, Gene Ontology; BP, Biological Process; CC, Cellular Component; MF, Molecular Function; CKD, Chronic kidney disease; HASMCs, Human aortic smooth muscle cells; HBSS, Hank’s Balanced Salt Solution; Pi, Inorganic phosphate; Ca, Calcium; qRT-PCR, Quantitative real-time polymerase chain reaction; STITCH, Search Tool for Interacting Chemicals.
