# Supplementary material for: Isowighteone attenuates vascular calcification by targeting HSP90AA1-mediated PI3K-Akt pathway and suppressing osteogenic gene expression
Source: Front Bioeng Biotechnol. 2025 Aug 20;13:1636883. doi: 10.3389/fbioe.2025.1636883 (PMC12405412; doi:10.3389/fbioe.2025.1636883)
Supplement: Supplementary file 6 [file Table6.docx]

Mechanism of isowighteone, a bioactive isoflavonoid constituent from *Ficus hispida* L.f., in the treatment of vascular calcification based on network pharmacology and experimental validation

Yuanxi Mo^1#^, Wanzi Hong^3#^, Zhaoyan Xu^3#^, Jiahui Peng^2^, Rui Yang^4^, Qiqi Song^4^, Yaoxin Liu^1^, Yuqi Cheng^4^, Wing-tak Wong^5^,Lei Jiang^1^*, An Jin^2^*, Ning Tan^1^*

*1.Guangdong Cardiovascular Institute, Guangdong Provincial People's Hospital,Guangdong Academy of Medical Science*

*2.School of Pharmaceutical Sciences, Hunan University of Medicine, Huaihua, 418000, China*

*3.Department of Cardiology, the Second School of Clinical Medicine, The First People Hospital of Foshan, Southern Medical University, Guangzhou, China.*

*4.School of Medicine South China University of Technology；Guangzhou；51006*

*5.Department of Applied Biology and Chemical Technology, The Hong Kong Polytechnic University, Hung Hom, Hong Kong, China.*

*Corresponding authors.

**Ning Tan**

Guangdong Cardiovascular Institute, Guangdong Provincial People's Hospital,Guangdong Academy of Medical Science, Guangzhou, 510006, China.

E-mail address: tanning100@126.com

**An Jin**

School of Pharmaceutical Sciences, Hunan University of Medicine, Huaihua, 418000, China

E-mail address: hnmujinan@163.com

**Lei Jiang**

Guangdong Cardiovascular Institute, Guangdong Provincial People's Hospital,Guangdong Academy of Medical Science, Guangzhou, 510006, China.

E-mail address: jianglei@smu.edu.cn

^#^These authors contributed equally to this work.

Supplementary materials

Table S1 Primer list

| Gene name | Sequence(5’-3’) | Annealing temperatures | Amplicon sizes |
| --- | --- | --- | --- |
| human-β-actin-F | CATGTACGTTGCTATCCAGGC | 59.13 | 250 |
| human-β-actin-R | CTCCTTAATGTCACGCACGAT | 58.46 |  |
| human-BMP2-F | TTCGGCCTGAAACAGAGACC | 61.8 | 83 |
| human-BMP2-R | CCTGAGTGCCTGCGATACAG | 62.3 |  |
| human-MSX2-F | TGGATGCAGGAACCCGG | 58.91 | 51 |
| human-MSX2-R | AGGGCTCATATGTCTTGGCG | 59.89 |  |
| human-RUNX2-F | GCCTTCCACTCTCAGTAAGAAGA | 59.49 | 68 |
| human-RUNX2-R | GCCTGGGGTCTGAAAAAGGG | 60.90 |  |
| human-HSP90AA1-F | GCTTGACCAATGACTGGGAAG | 59.18 | 187 |
| human-HSP90AA1-R | AGCTCCTCACAGTTATCCATGA | 58.62 |  |
| mouse-HSP90AA1-F | GACGCTCTGGATAAAATCCGTT | 60.4 | 88 |
| mouse-HSP90AA1-R | TGGGAATGAGATTGATGTGCAG | 60.4 |  |
| mouse-β-actin-F | GGCTGTATTCCCCTCCATCG | 59.96 | 154 |
| mouse-β-actin-R | CCAGTTGGTAACAATGCCATGT | 59.44 |  |

Table S2 The intersection of VC and isowighteone target gene

| Gene | ID | Gene | ID | Gene | ID |
| --- | --- | --- | --- | --- | --- |
| EGFR | [P00533](https://www.uniprot.org/uniprotkb/P00533/entry) | CYP19A1 | [P11511](https://www.uniprot.org/uniprotkb/P11511/entry) | ESRRA | [P11474](https://www.uniprot.org/uniprotkb/P11474/entry) |
| F2 | [P00734](https://www.uniprot.org/uniprot/P00734) | BCL2L1 | [Q07817](https://www.uniprot.org/uniprotkb/Q07817/entry) | ESRRB | [O9571](https://www.uniprot.org/uniprotkb/O95718/entry)8 |
| ESR1 | [P03372](https://www.uniprot.org/uniprotkb/P03372/entry) | ABCB1 | [P08183](https://www.uniprot.org/uniprotkb/P08183/entry) | ADORA1 | [P30542](https://www.uniprot.org/uniprotkb/P30542/entry) |
| ALPL | [P05186](https://www.uniprot.org/uniprotkb/P05186/entry) | LRRK2 | [Q5S007](https://www.uniprot.org/uniprotkb/Q5S007/entry) | RPS6KA3 | [P51812](https://www.uniprot.org/uniprotkb/P51812/entry) |
| CSF1R | [P07333](https://www.uniprot.org/uniprotkb/P07333/entry) | CDK2 | [P24941](https://www.uniprot.org/uniprotkb/P24941/entry) | CA12 | [P02458](https://www.uniprot.org/uniprotkb/P02458/entry) |
| PLAU | [P00749](https://www.uniprot.org/uniprotkb/P00749/entry) | IKBKB | O14920 | MAK | [P20794](https://www.uniprot.org/uniprotkb/P20794/entry) |
| KIT | [P10721](https://www.uniprot.org/uniprotkb/P10721/entry) | MAOB | [P27338](https://www.uniprot.org/uniprotkb/P27338/entry) | DRD4 | [P21917](https://www.uniprot.org/uniprotkb/P21917/entry) |
| PON1 | [P27169](https://www.uniprot.org/uniprotkb/P27169/entry) | TYK2 | [P29597](https://www.uniprot.org/uniprotkb/P29597/entry) | SLC6A2 | [P23975](https://www.uniprot.org/uniprotkb/P23975/entry) |
| MAPK8 | [P45983](https://www.uniprot.org/uniprotkb/P45983/entry) | ADRB2 | [P07550](https://www.uniprot.org/uniprotkb/P07550/entry) | HTR2C | [P28335](https://www.uniprot.org/uniprotkb/P28335/entry) |
| BCL2 | [P10415](https://www.uniprot.org/uniprotkb/P10415/entry) | ALDH2 | [P05091](https://www.uniprot.org/uniprotkb/P05091/entry) | CDKL5 | [O76039](https://www.uniprot.org/uniprotkb/O76039/entry) |
| PDGFRA | [P16234](https://www.uniprot.org/uniprotkb/P16234/entry) | ADORA2A | [P29274](https://www.uniprot.org/uniprotkb/P29274/entry) | WEE1 | [P30291](https://www.uniprot.org/uniprotkb/P30291/entry) |
| SIRT1 | [Q96EB6](https://www.uniprot.org/uniprotkb/Q96EB6/entry) | CA4 | P22748 | HSD17B1 | [P14061](https://www.uniprot.org/uniprotkb/P14061/entry) |
| RAF1 | [P04049](https://www.uniprot.org/uniprotkb/P04049/entry) | EPHA2 | [P29317](https://www.uniprot.org/uniprotkb/P29317/entry) | CDC25B | [P30305](https://www.uniprot.org/uniprotkb/P30305/entry) |
| ESR2 | [Q92731](https://www.uniprot.org/uniprotkb/Q92731/entry) | ACHE | [Q04844](https://www.uniprot.org/uniprotkb/Q04844/entry) | MELK | [Q14680](https://www.uniprot.org/uniprotkb/Q14680/entry) |
| XDH | [P47989](https://www.uniprot.org/uniprotkb/P47989/entry) | PTPN1 | [P18031](https://www.uniprot.org/uniprotkb/P18031/entry) |  |  |
| EP300 | [Q09472](https://www.uniprot.org/uniprotkb/Q09472/entry) | DRD2 | [P14416](https://www.uniprot.org/uniprotkb/P14416/entry) |  |  |
| ABL1 | [P00519](https://www.uniprot.org/uniprotkb/P00519/entry) | DYRK1A | [Q13627](https://www.uniprot.org/uniprotkb/Q13627/entry) |  |  |
| IL2 | [P60568](https://www.uniprot.org/uniprotkb/P60568/entry) | ABCG2 | [Q9UNQ0](https://www.uniprot.org/uniprotkb/Q9UNQ0/entry) |  |  |
| HSP90AA1 | P07900 | TYR | [P17643](https://www.uniprot.org/uniprotkb/P17643/entry) |  |  |
| NOX4 | [Q9NPH5](https://www.uniprot.org/uniprotkb/Q9NPH5/entry) | JAK3 | [P24394](https://www.uniprot.org/uniprotkb/P24394/entry) |  |  |
| MIF | [P14174](https://www.uniprot.org/uniprotkb/P14174/entry) | OPRM1 | Q15363 |  |  |
| HTR2A | [P28223](https://www.uniprot.org/uniprotkb/P28223/entry) | ALOX15 | P30086 |  |  |
| MAOA | [P21397](https://www.uniprot.org/uniprotkb/P21397/entry) | GRIN2B | Q[13224](https://www.uniprot.org/uniprotkb/Q13224/entry) |  |  |
| ALOX12 | [P18054](https://www.uniprot.org/uniprotkb/P18054/entry) | CHEK1 | [O14757](https://www.uniprot.org/uniprotkb/O14757/entry) |  |  |
| CDK4 | [P11802](https://www.uniprot.org/uniprotkb/P11802/entry) | TBXAS1 | [P24557](https://www.uniprot.org/uniprotkb/P24557/entry) |  |  |

Table S3:String_node_degrees

Table S4: Details of BP domain

Table S5:Details of CC domain

Table S6:Details of MF domain

Table S7:Details of KEGG analysis
